# An Assessment of Attendee Experiences With a Workshop to Reframe Aging-Related Communications

**DOI:** 10.1093/geroni/igaa057.3311

**Published:** 2020-12-16

**Authors:** Stephanie Chesser, Michelle Porter

**Affiliations:** University of Manitoba, Winnipeg, Manitoba, Canada

## Abstract

The Centre on Aging at the University of Manitoba adapted the FrameWorks Institute’s Gaining Momentum toolkit into an interactive workshop that was delivered to aging-related stakeholders (e.g., health professionals, educators, researchers, advocates, older persons) across the province of Manitoba, Canada. The purpose of this study was to complete a qualitative assessment of attendee experiences with the workshop and its impact, if any, on their aging-related communication behaviors. Study participants recruited from four communities (two urban, two rural) completed two telephone interviews—one approximately two weeks and one approximately three months post-workshop—about their experiences, motivations for attending, and short and long-term communication goal setting. Through the interviews, most participants expressed positive experiences with the workshop content, as well as an enjoyment of its interactive elements (e.g., self-introduction activity, reframing short and long form aging-related communications, group feedback). Several also shared specific examples of how workshop content was influencing their short/long-term aging communication strategies. Some participants, however, also identified ways that local culture could impact the interpretation of and, thus, potential success of workshop language and/or framing recommendations in specific communities. Overall, the findings from this study suggest that the Gaining Momentum workshop was a valuable experience for attendees that inspired critical assessment of, and changes to, the ways they communicated about aging in their professional and personal lives. Future research is warranted to explore the ways its content could be adapted to better meet the unique communication considerations within the province of Manitoba (e.g., cultural, geographic, and language-related implications).

